# Myocardial bridging of the left anterior descending coronary artery is associated with reduced myocardial perfusion reserve: a ^13^N-ammonia PET study

**DOI:** 10.1007/s10554-018-1460-8

**Published:** 2018-09-28

**Authors:** Andrea G. Monroy-Gonzalez, Erick Alexanderson-Rosas, Niek H. J. Prakken, Luis E. Juarez-Orozco, Lourdes Walls-Laguarda, Enrique A. Berrios-Barcenas, Aloha Meave-Gonzalez, Jan C. Groot, Riemer H. J. A. Slart, Rene A. Tio

**Affiliations:** 1 Medical Imaging Center, University Medical Center Groningen, University of Groningen, Groningen, The Netherlands; 2 Department of Cardiology, Catharina Hospital, Eindhoven, The Netherlands; 3 National Institute of Cardiology Ignacio Chavez, Mexico City, Mexico; 4 Biomedical Photonic Imaging, Faculty of Science and Technology, University of Twente, Enschede, The Netherlands; 5 Department of Physiology, National Autonomous University of Mexico, Mexico City, Mexico

**Keywords:** Myocardial bridging, Microvascular dysfunction, Myocardial perfusion, Positron emission tomography, Coronary computed tomography angiography

## Abstract

Myocardial Bridging (MB) refers to the band of myocardium that abnormally overlies a segment of a coronary artery. This paper quantitatively evaluates the influence of MB of the left anterior descending artery (LAD) on myocardial perfusion of the entire left ventricle. We studied 131 consecutive patients who underwent hybrid rest/stress ^13^N-ammonia positron emission tomography (PET) and coronary computed tomography angiography (CCTA) due to suspected myocardial ischemia. Patients with previous myocardial infarction and/or significant coronary artery disease (≥ 50% stenosis) were excluded. Myocardial perfusion measurements were compared between patients with and without LAD-MB. Additionally, we evaluated the relationship between anatomical characteristics (length and depth) of LAD-MB and myocardial perfusion measurements. 17 (13%) patients presented a single LAD-MB. Global myocardial perfusion reserve (MPR) was lower in patients with LAD-MB than in patients without LAD-MB (1.9 ± 0.5 vs. 2.3 ± 0.6, p < 0.01). Global stress myocardial blood flow (MBF) was similar in patients with and without LAD-MB (2.2 ± 0.4 vs. 2.3 ± 0.7 ml/g/min, p = 0.40). Global rest MBF was higher in patients with LAD-MB than in patients without LAD-MB (1.2 ± 0.3 vs. 1.0 ± 0.2 ml/g/min, p < 0.01). Global rest MBF, stress MBF, and MPR quantifications were similar in patients with superficial and deep LAD-MB (all p = NS). We did not find any correlation between length and global rest MBF, stress MBF nor MPR (r = − 0.14, p = 0.59; r = 0.44, p = 0.07; and r = 0.45, p = 0.07 respectively). Quantitative myocardial perfusion suggests that LAD-MB may be related to impaired perfusion reserve, an indicator of microvascular dysfunction. Anatomical characteristics of LAD-MB were not related to changes in myocardial perfusion.

**Electronic supplementary material:**

The online version of this article (10.1007/s10554-018-1460-8) contains supplementary material, which is available to authorized users.

## Introduction

Myocardial bridging (MB) refers to the band of myocardium that abnormally overlies a segment of a coronary artery, which regularly (70–98%) concerns the left anterior descending artery (LAD) [1]. The clinical presentation of this coronary anomaly varies widely from asymptomatic patients to those with myocardial ischemia, myocardial infarction, and even sudden death [2–6].

Until now the clinical significance of MB remains unclear. Invasive studies have revealed that patients with severe epicardial compression of the LAD-MB may show anterior wall subendocardial or transmural ischemia [1, 7, 8]. It has also been reported that patients with mild compression of the LAD-MB may show a normal distal flow of the LAD and septal ischemia, also called “branch steal phenomenon”, due to high velocity within the MB segment and changes in perfusion pressure affecting septal branches of the LAD [9, 10].

The existing body of research suggests that visual and physiological assessment can help explain the pathophysiology of MB. Cardiac hybrid imaging with positron emission tomography (PET)/computed tomography (CT) is a validated technique that allows anatomical and functional evaluation of the heart [11, 12]. It is widely accepted that low values of stress myocardial blood flow (MBF) and/or myocardial perfusion reserve (MPR) measured by PET can determine the hemodynamic significance of a stenosis, while these measurements can also assess microvascular function in the absence of epicardial stenosis [12, 13]. On the other hand, coronary CT angiography (CCTA) has increased the detection of MB with precise delineation of anatomical characteristics, including location, depth, length, systolic compression, and concurrent presence of atherosclerosis [1].

Currently, myocardial perfusion consequences of MB are not fully understood. While it has been suggested that MB may relate to perfusion defects, only one study has reported that MB is not associated with distal abnormal stress MBF. It is unknown whether LAD-MB is related to abnormal perfusion quantifications in the entire vascular territory of the LAD or in other regions of the left ventricle. It has also been hypothesized that anatomical characteristics of a LAD-MB, especially depth and length, are related to the clinical presentation, but this remains unclear [14].

This paper evaluates the influence of LAD-MB on myocardial perfusion of the entire left ventricle. There were three aims of this study: firstly, to compare global perfusion measurements of patients with and without LAD-MB; secondly, to compare regional perfusion measurements of the three vascular territories (LAD, LCx, and RCA) of patients with and without LAD-MB; thirdly, to evaluate the relationship of anatomical characteristics (length and depth) of LAD-MB, measured by CCTA, and quantitative perfusion measurements, as measured by PET.

## Methods

### Patient population and study design

We retrospectively studied 131 consecutive patients between January 2010 and March 2016. All patients, suspected of coronary artery disease (CAD), were referred for ^13^N-ammonia PET myocardial perfusion imaging and CCTA, at the PET/CT Unit of the National Autonomous University of Mexico, as part of the clinical workup. MB patients were diagnosed based on their CCTA results. Exclusion criteria for both patients with and without MB were previous myocardial infarction, significant CAD (≥ 50% stenosis) detected by CCTA, non-ischemic cardiomyopathy, and/or other congenital coronary abnormalities. All patients signed an informed consent to undergo ^13^N-ammonia PET/CCTA. The institutional ethics committee approved the conduction of the study.

### PET myocardial perfusion

All scans were acquired with a PET/CT scanner (Biograph True Point PET/CT 64-Multislice Scanner; Siemens Medical, Erlangen, Germany). Patients had an overnight fast and were refrained from caffeine and theophylline for 24 h before the study. Myocardial PET data were acquired at rest and during adenosine stress, as previously described [15]. In brief, 10 min rest imaging acquisition started with a 740 MBq of ^13^N-ammonia i.v. injection. After 30 min, pharmacological stress was performed with an i.v. injection of adenosine during a 6-min period (140 µg/kg/min). A second dose of 740 MBq of ^13^N-ammonia was injected i.v. at the third minute of the pharmacological stress and imaging acquisition started few seconds before the radiotracer injection for a duration of 10 min. Static, dynamic, and gated datasets were obtained at rest and stress and further analyzed with automated QPET software v16 (Cedars Sinai, LA, USA) [16].

An expert nuclear cardiologist (E.A.R.) analyzed the perfusion images. Dynamic data was used to perform quantification of rest and stress MBF, which are expressed in milliliter/gram/minute (ml/g/min) of myocardial tissue. Because rest MBF depends on cardiac workload, rest MBF was corrected for the rate pressure product (RPP) (rest MBF/RPP × 1000) [17, 18]. MPR was quantified as the ratio of absolute stress MBF to the corrected rest MBF. Rest MBF, stress MBF, and MPR were analyzed globally and regionally according to the three vascular territories using the automatic grid function “Vessels”. Abnormal absolute MBF during stress with adenosine and abnormal MPR were considered as < 1.9 ml/g/min MPR < 2.0, respectively [19]. Semi-quantification of myocardial perfusion imaging was performed on static images of the 17 segments of the heart [20]. Each segment was reported as 0 = normal uptake, 1 = mildly reduced, 2 = moderate reduced, 3 = severely reduced, and 4 = absence of uptake [15]. Summed scores are reported as summed stress score (SSS), summed rest score (SRS), and summed difference score (SDS) [15]. An SSS ≥ 4 was considered as abnormal. Also left ventricular ejection fraction (LVEF) was assessed using gated datasets during rest and stress and analyzed automatically by the software.

### Coronary computed tomography angiography

CCTA scans followed the PET acquisition. Beta-blockers were administered if the heart rate was > 65 beats per minute. Sublingual short-acting nitrates were administrated 3–4 min prior to the scan. A CCTA contrast-enhanced scan was obtained after the administration of 60–80 mL of Iopamiron 370 IV (rate 5 mL/s) during a single breath-hold (10 s). The scan was performed with the following parameters: 0.60 mm collimation, gantry rotation of 330 ms, a pitch of 0.2, X-ray tube current of 550–945 mA, and a tube voltage of 120 kVp. A non-contrast low dose radiation scan was used to determine coronary artery calcium (CAC) score. To obtain motion-free images standard multiphase prospective, electrocardiography-gated, and half-scan reconstruction windows were centered on the best cardiac phase image for analysis.

One experienced radiologist (A.M.G.) and one experienced cardiologist (E.A.B.B.) specialized in cardiovascular CCTA processed the studies using a dedicated Leonardo workstation (Siemens Medical Systems, Erlangen, Germany). CCTA raw datasets were reconstructed automatically by Syngo Via software (Siemens Medical Systems, TN, USA), as previously described [15]. Global CAC was reported in Agatston Units (AU). Readers assessed the presence and the degree of coronary artery stenosis in the proximal, middle, and distal segments of the LAD, Left Circumflex Coronary Artery (LCx) and Right Coronary Artery (RCA), as well as their major branches. The presence of non-obstructive CAD is referred to the presence of < 50% stenosis. Vessels with < 2 mm of diameter were excluded from the analysis. MB was defined as the complete intramyocardial course of a coronary artery, including thin MB [1]. MB localized at a depth of < 2 mm and ≥ 2 mm were considered superficial and deep respectively [14].

### Statistical methods

Categorical variables are presented as simple proportions. Chi square and Fisher tests were used to compare proportions of variables. Parametric data were presented as the mean ± standard deviation and were compared using Student t-test. Non-parametric data were presented as the median and interquartile range (IQR) and were compared using the Mann–Whitney *U* test. Linear regression analysis was used to determine the relationship between variables. Stepwise forward selection was used to create a multiple linear regression model. We used the Spearman product-moment correlation coefficient (r) to describe the strength and direction of the correlations between continuous variables. A 2-tailed p-value ≤ 0.05 was considered statistically significant. All statistical analyses were performed using SPSS v23.

## Results

In our population, 17 patients presented a single LAD-MB. Baseline characteristics of patients with and without LAD-MB were similar (Table 1).

**Table 1:** Baseline Characteristics of Patients with and without LAD-MB

	Patients with LAD-MB (n = 17)	Patients without LAD-MB (n = 114)	p value
Age	63 ± 11	60 ± 11	0.28
Male gender	7 (47)	70 (63)	0.27
Hypertension	9 (60)	62 (58)	0.99
DM type 2	1 (7)	18 (17)	0.30
Dyslipidemia	7 (47)	62 (58)	0.42
Current smoker	4 (29)	46 (43)	0.53
BMI	29 ± 5	28 ± 5	0.38
LVEF rest (%)	67 ± 11	69 ± 7	0.60
LVEF stress (%)	69 ± 10	70 ± 7	0.81
Rate pressure product in rest	7857 ± 1932	8258 ± 1944	0.43
Rate pressure product in stress	10,302 ± 2579	9450 ± 2501	0.26

Values are mean ± standard deviation, n (%)

*BMI* body mass index, *DM* diabetes mellitus, *LAD* left anterior descending artery, *LVEF* left ventricular ejection fraction, *MB* myocardial bridging

### Global perfusion analysis

Global quantitative myocardial perfusion comparisons are shown in Table 2. Mean global rest MBF was significantly increased in LAD-MB patients. Mean global stress MBF was similar in patients with and without LAD-MB. Mean global MPR was significantly lower in patients with a LAD-MB than in patients without MB.

**Table 2:** Comparison of quantitative perfusion measurements between LAD-MB and patients without LAD-MB

Variable	Patients with LAD-MB (n = 17)	Patients without LAD-MB (n = 114)	p value
Global rest MBF (ml/g/min)	1.2 ± 0.3	1.0 ± 0.2	0.002
Global stress MBF (ml/g/min)	2.2 ± 0.4	2.3 ± 0.7	0.42
Global MPR	1.9 ± 0.5	2.3 ± 0.6	0.001

Values are shown as mean ± standard deviation

*LAD* left anterior descending artery, *MB* myocardial bridging, *MBF* myocardial blood flow, *MPR* myocardial perfusion reserve

It was observed that the proportion of patients with an abnormal MPR (< 2.0) was increased in LAD-MB patients [70% (n = 12) vs. 30% (n = 34), p = 0.01]. However, the proportion of patients with an abnormal stress MBF (< 1.9) was similar in patients with and without an LAD-MB [12% (n = 2) vs. 24% (n = 27), p = 0.36]. Multiple linear regression analysis demonstrated that among cardiovascular risk factors only age and LAD-MB predicted a decrease in MPR in our study population (Table 3). Multiple linear regression analysis also demonstrated that male gender, dyslipidaemia, and body mass index predicted a decrease in stress MBF in our study population (Online Resource 1).

**Table 3:** Univariate and multiple linear regression analyses showing possible predictors of MPR

	Univariate linear regression ß (95% CI)	R^2^	p value	Multiple linear regression ß (95% CI)	p value	R^2^
Age	− 0.01 (− 0.02 to 0.00)	0.03	0.04	0.22 (− 0.02 to − 0.001)	0.03	0.11
Male gender	0.11 (− 0.12 to 0.34)	0.01	0.33			
Hypertension	− 0.05 (− 0.27 to 0.18)	0.00	0.68			
Dyslipidaemia	− 0.04 (− 0.27 to 0.19)	0.00	0.74			
DM type 2	0.19 (− 0.10 to 0.49)	0.01	0.20	0.22 (− 0.07 to 0.51)	0.14	
Current smoker	− 0.03 (− 0.18 to 0.12)	0.00	0.68			
BMI	− 0.01 (− 0.03 to 0.02)	0.00	0.57			
LVEF in rest	− 0.01 (− 0.02 to 0.01)	0.00	0.34			
LVEF in stress	− 0.01 (− 0.02 to 0.01)	0.00	0.46			
Calcium score	− 0.001 (− 0.002 to 0.001)	0.01	0.47			
Presence of non-significant CAD	− 0.09 (− 0.32 to 0.11)	0.01	0.41			
LAD-MB	− 0.49 (− 0.81 to − 0.17)	0.07	0.003	− 0.42 (− 0.75 to − 0.10)	0.01	

Age, DM type 2, and MB are entered in the multiple linear regression model

*BMI* body mass index, *CAD* coronary artery disease, *DM* diabetes mellitus, *LAD* left anterior descending artery, *LVEF* left ventricular ejection fraction, *MB* myocardial bridging

The proportion of patients with an abnormal SSS (≥ 4) was larger in patients with a MB in comparison to patients without MB [65% (n = 11) vs. 39% (n = 45), p = 0.05]. However, SSS did not reveal any differences between patients with and without LAD-MB [5 (IQR 1–8) vs. 2 (IQR 0–5), p = 0.07]. Also, SRS was similar in patients with and without LAD-MB [0 (IQR 0–1) vs. 0 (IQR 0–1), p = 0.50]. A significant increase in SDS was observed in patients with LAD-MB [5 (IQR 1–7) vs. 1 (IQR 0–5), p = 0.02].

Presence of non-significant CAD was similar in patients with and without LAD-MB [41% (n = 7) vs. 44% (n = 50), p = 0.83]. CAC was similar in patients with and without LAD-MB [0 (IQR 0–16) vs. 0 (IQR 0–37), p = 0.61].

### Regional perfusion analysis

Regional quantitative perfusion comparisons are summarized in Table 4. Rest MBF was significantly increased in the three vascular territories of MB patients. Stress MBF measurements were similar in patients with and without LAD-MB in the in the three vascular territories. MPR was decreased in all three vascular territories of MB patients.

**Table 4:** Comparison of regional quantitative perfusion measurements between LAD-MB and patients without LAD-MB

Variable	Patients with LAD-MB (n = 17)	Patients without LAD-MB (n = 114)	p value
LAD			
Rest MBF (ml/g/min)	1.2 ± 0.3	1.0 ± 0.2	0.005
Stress MBF (ml/g/min)	2.3 ± 0.5	2.4 ± 0.7	0.73
MPR	2.0 ± 0.4	2.4 ± 0.7	0.001
LCx			
Rest MBF (ml/g/min)	1.3 ± 0.3	1.1 ± 0.3	0.001
Stress MBF (ml/g/min)	2.3 ± 0.5	2.4 ± 0.7	0.61
MPR	1.8 ± 0.4	2.4 ± 0.7	< 0.001
RCA			
Rest MBF (ml/g/min)	1.1 ± 0.3	1.0 ± 0.3	0.02
Stress MBF (ml/g/min)	1.8 ± 0.5	2.1 ± 0.7	0.13
MPR	1.7 ± 0.8	2.2 ± 0.7	0.006

Values are mean ± standard deviation

*LAD* left anterior descending artery, *LCx* left circumflex artery, *MB* myocardial bridging, *MBF* myocardial blood flow, *MPR* myocardial perfusion reserve, *RCA* right coronary artery

SSS was higher in the region of the LCx in patients with LAD-MB [2 (IQR 0–5) vs. 0 (IQR 0–2), p = 0.04]. SDS was also higher in the region of the LCx in patients with LAD-MB [0 (IQR 0–5) vs. 0 (IQR 0–2), p = 0.01]. Remaining regional SRS, SSS, and SDS were similar between patients with and without LAD-MB (Online Resource 2).

Presence of non-significant CAD was similar in patients with and without LAD-MB in the three coronary territories [LAD: 35% (n = 6) vs. 22% (n = 26), p = 0.36; LCx: 29% (n = 5) vs. 39% (n = 44), p = 0.47; RCA: 29% (n = 5) vs. 27% (n = 31), p = 0.99]. CAC was similar in patients with and without LAD-MB in the three coronary territories [LAD: 0 (IQR 0–2) vs. 0 (IQR 0–3), p = 0.87; LCx: 0 (IQR 0–0) vs. 0 (IQR 0–0), p = 0.87; RCA: 0 (IQR 0–0) vs. 0 (IQR 0–2), p = 0.38].

### Anatomical characteristics of LAD-MB

Anatomical characteristics of LAD-MB are presented in the Online Resource 3. In patients with LAD-MB, we did not find a correlation between length and quantitative perfusion measurements, both global and regional (Online Resource 4). Also, global and regional rest MBF, stress MBF, and MPR were similar in patients with superficial and deep LAD-MB (Online Resource 5).

## Discussion

The main findings of this study are that: (1) LAD-MB is related to a decreased global MPR; (2) decrease of MPR can be measured in the three coronary territories; (3) anatomical characteristics of LAD-MB did not have an effect on myocardial perfusion.

### Global perfusion analysis

This study shows that the presence of LAD-MB is related to a low MPR, an indicator of microvascular dysfunction, as a result of a high global rest MBF. In our study, patients with LAD-MB showed 17% less global MPR than patients without LAD-MB. To our knowledge, there are no previous results reporting global quantitative myocardial perfusion differences in patients with MB. A decreased MPR in the presence of high rest MBF has been reported in patients with chest pain and normal coronary arteries, hypertension, or obesity [21, 22]. One remaining question is whether a low MPR implies an increased cardiovascular risk in LAD-MB. A recent meta-analysis has shown that MB is related to major adverse cardiac events and ischemia, however, the stratification of patients with an MB remains a challenge [5]. Meanwhile, it is well known that MPR is a marker of microvascular health in patients without obstructive coronary artery disease [23]. Gupta et al. reported that a reduced MPR, even in the presence of normal stress MBF and a high rest MBF, is linked to adverse outcomes [24]. Therefore, it is possible that MPR measured by PET may help to identify MB patients that have an increased cardiovascular risk due to microvascular dysfunction. However, prospective studies are needed to confirm our results in a larger population and to determine the prognostic value of stress MBF and MPR in patients with MB.

### Regional perfusion analysis

**Fig. 1:**
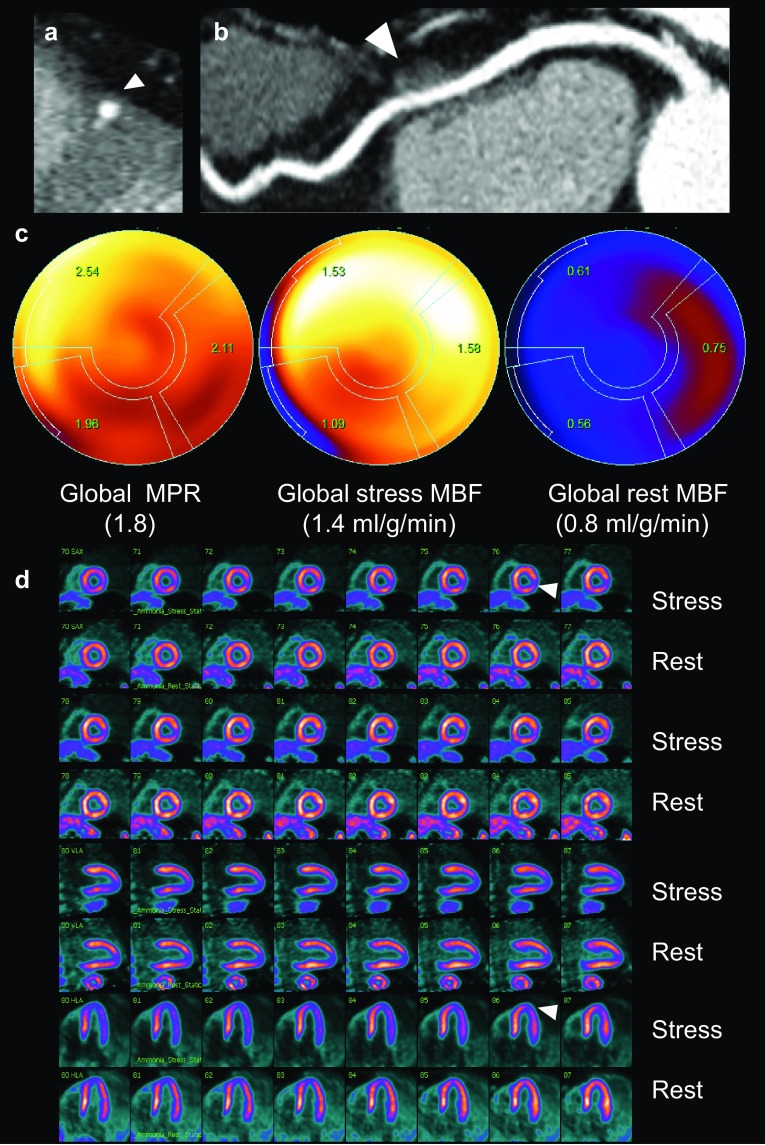
**a, b** CCTA demonstrated a LAD-MB (*white arrows*) and no coronary artery disease in the LAD, LCx, nor RCA. **c** Dynamic polar map of ^13^N-ammonia PET shows low global MPR (< 2.0) and low global stress MBF (< 1.9 ml/g/min). **d** Myocardial perfusion imaging showed apical and inferolateral mild ischemia (*white arrows*)

In the presence of LAD-MB, we found that the decrease of MPR in the three vascular territories (LAD, LCx, and RCA) is related to an increased rest MBF rather than to a decreased stress MBF. These results indicate that the presence of a LAD-MB may coexist with a state of microvascular dysfunction of the left ventricle, which might explain why myocardial perfusion defects, including those in the anterior, lateral and inferior walls, are more common in patients with LAD-MB (Fig. 1) [25, 26]. Interestingly, we did not find an increase of perfusion defects in the LAD territory that indicate anterior or septal ischemia in patients with a LAD-MB, as suggested before [9]. Our results support that the decreased of MPR in patients with LAD-MB is not the result of a flow-limiting significance of the MB since stress MBF, which is an accurate parameter of stenosis, was similar in patients with and without LAD-MB [27]. Similar to our study, Uusitalo et al. first reported the relation between MB and absolute rest and stress MBF in the distal segments affected by an MB of any coronary artery, using adenosine stress and Oxygen-15 water PET, without finding any decreased stress MBF in the MB affected territory [28]. Because of heterogeneity of methodology and results in previous studies, which mostly focus on one coronary territory, we suggest that global and regional quantitative assessment of the three vascular territories could help better to understand the clinical presentation of MB.

### Anatomical characteristics of LAD-MB

Our study did not show differences in perfusion quantifications in patients with a deep and superficial LAD-MB. Also, the length of LAD-MB did not correlate with myocardial perfusion quantifications. However, with a small sample size, caution must be applied, as possibility remains that other findings could influence hemodynamics, such as the location of MB in the segment of the LAD and diameter of stenosis [10, 29]. In this setting, dobutamine stress might play an important role in determining the significance of LAD-MB by better delineating the severity of MB compression. Therefore, further work could still establish a relationship between quantitative myocardial perfusion and MB anatomical characteristics including precise location, length, depth, and compression of an MB.

### Study limitations

Our study has some limitations due to its retrospective nature. We studied a discrete population of patients with LAD-MB, however, similar prevalence has been previously reported [2]. Furthermore, because we studied patients with suspected CAD, we cannot extrapolate our results to asymptomatic subjects. Another limitation is that we were not able to fully analyze the hemodynamic severity of compression of the intramyocardial segments produced during systole due to a prospective triggering of the CCTA and an adenosine stress [2, 10].

## Conclusions

In our study, quantitative perfusion assessment by PET suggests that LAD-MB may be related to impaired perfusion reserve, an indicator of microvascular dysfunction. Anatomical characteristics of LAD-MB were not related to changes in myocardial perfusion. Global and regional quantitative myocardial perfusion assessment is a promising tool that may help to better understand the clinical significance of LAD-MB and to identify patients who may benefit from further therapies. Prospective studies are needed to confirm our results and to determine the prognostic value of stress MBF and MPR in patients with MB.

## Electronic supplementary material

Below is the link to the electronic supplementary material.

Supplementary material 1 (DOCX 18 KB)

Supplementary material 2 (DOCX 16 KB)

Supplementary material 3 (DOCX 18 KB)

Supplementary material 4 (DOCX 17 KB)

Supplementary material 5 (DOCX 17 KB)

## Abbreviations

MB: Myocardial bridging
LAD: Left anterior descending artery
LCx: Left circumflex coronary artery
RCA: Right coronary artery
PET: Positron emission tomography
CCTA: Coronary computed tomography angiography
MPR: Myocardial perfusion reserve
MBF: Myocardial blood flow
CAD: Coronary artery disease
SSS: Summed stress score
SRS: Summed rest score
SDS: Summed difference score
CAC: Coronary artery calcium score

## References

[1] Tarantini G, Migliore F, Cademartiri F (2016). Left anterior descending artery myocardial bridging. J Am Coll Cardiol.

[2] Nakanishi R, Rajani R, Ishikawa Y (2012). Myocardial bridging on coronary CTA: an innocent bystander or a culprit in myocardial infarction?. J Cardiovasc Comput Tomogr.

[3] Corban MT, Hung OY, Eshtehardi P (2014). Myocardial bridging. J Am Coll Cardiol.

[4] Tio RA, Van Gelder IC, Boonstra PW, Crijns HJ (1997). Myocardial bridging in a survivor of sudden cardiac near-death: role of intracoronary doppler flow measurements and angiography during dobutamine stress in the clinical evaluation. Heart.

[5] Hostiuc S, Rusu MC, Hostiuc M (2017). Cardiovascular consequences of myocardial bridging: a meta-analysis and meta-regression. Sci Rep.

[6] Hazenberg AJC, Jessurun GAJ, Tio RA (2008). Mechanisms involved in symptomatic myocardial bridging. Neth Heart J.

[7] Gould KL, Johnson NP (2015). Myocardial bridges: lessons in clinical coronary pathophysiology. JACC Cardiovasc Imaging.

[8] Escaned J, Cortés J, Flores A (2003). Importance of diastolic fractional flow reserve and dobutamine challenge in physiologic assessment of myocardial bridging. J Am Coll Cardiol.

[9] Lin S, Tremmel JA, Yamada R (2013). A novel stress echocardiography pattern for myocardial bridge with invasive structural and hemodynamic correlation. J Am Heart Assoc.

[10] Gould KL, Kirkeeide R, Johnson NP (2010). Coronary branch steal experimental validation and clinical implications of interacting stenosis in branching coronary arteries. Circ Cardiovasc Imaging.

[11] Slart RHJA, Glauche J, Golestani R (2012). PET and MRI for the evaluation of regional myocardial perfusion and wall thickening after myocardial infarction. Eur J Nucl Med Mol Imaging.

[12] Dorbala S, Di Carli MF (2014). Cardiac PET perfusion: prognosis, risk stratification, and clinical management. Semin Nucl Med.

[13] Camici PG, D’Amati G, Rimoldi O (2015). Coronary microvascular dysfunction: mechanisms and functional assessment. Nat Rev Cardiol.

[14] Jodocy D, Aglan I, Friedrich G (2010). Left anterior descending coronary artery myocardial bridging by multislice computed tomography: correlation with clinical findings. Eur J Radiol.

[15] Alexánderson Rosas E, Slomka PJ, García-Rojas L (2010). Functional impact of coronary stenosis observed on coronary computed tomography angiography: comparison with 13N-ammonia PET. Arch Med Res.

[16] Juárez-Orozco LE, Alexanderson E, Dierckx RA (2016). Stress myocardial blood flow correlates with ventricular function and synchrony better than myocardial perfusion reserve: a nitrogen-13 ammonia PET study. J Nucl Cardiol.

[17] Czernin J, Muller P, Chan S (1993). Influence of age and hemodynamics on myocardial blood flow and flow reserve. Circulation.

[18] Sciagrà R, Passeri A, Bucerius J (2016). Clinical use of quantitative cardiac perfusion PET: rationale, modalities and possible indications. Position paper of the Cardiovascular Committee of the European Association of Nuclear Medicine (EANM). Eur J Nucl Med Mol Imaging.

[19] Gould KL, Johnson NP, Bateman TM (2013). Anatomic versus physiologic assessment of coronary artery disease: role of coronary flow reserve, fractional flow reserve, and positron emission tomography imaging in revascularization decision-making. J Am Coll Cardiol.

[20] Cerqueira MD, Weissman NJ, Dilsizian V (2002). Standardized myocardial segmentation and nomenclature for tomographic imaging of the heart. J Cardiovasc Magn Reson.

[21] Graf S, Khorsand A, Gwechenberger M (2006). Myocardial perfusion in patients with typical chest pain and normal angiogram. Eur J Clin Invest.

[22] Cho S-G, Kim JH, Cho JY (2015). Characteristics of anginal patients with high resting myocardial blood flow measured with N-13 ammonia PET/CT. Nucl Med Commun.

[23] Murthy VL, Naya M, Taqueti VR (2014). Effects of sex on coronary microvascular dysfunction and cardiac outcomes. Circulation.

[24] Gupta A, Taqueti VR, van de Hoef TP (2017). Integrated noninvasive physiological assessment of coronary circulatory function and impact on cardiovascular mortality in patients with stable coronary artery disease. Circulation.

[25] Gawor R, Kuśmierek J, Płachcińska A (2011). Myocardial perfusion GSPECT imaging in patients with myocardial bridging. J Nucl Cardiol.

[26] Tang K, Wang L, Shi R (2011). The role of myocardial perfusion imaging in evaluating patients with myocardial bridging. J Nucl Cardiol.

[27] Schindler TH (2015). Myocardial blood flow: putting it into clinical perspective. J Nucl Cardiol.

[28] Uusitalo V, Saraste A, Pietilä M (2015). The functional effects of intramural course of coronary arteries and its relation to coronary atherosclerosis. JACC Cardiovasc Imaging.

[29] Zhang J-M, Zhong L, Luo T (2014). Numerical simulation and clinical implications of stenosis in coronary blood flow. Biomed Res Int.

