# Supplementary material for: Myocardial bridging of the left anterior descending coronary artery is associated with reduced myocardial perfusion reserve: a 13N-ammonia PET study
Source: Int J Cardiovasc Imaging. 2018 Sep 28;35(2):375–82. doi: 10.1007/s10554-018-1460-8 (PMC6428791; doi:10.1007/s10554-018-1460-8)
Supplement: Supplementary file 1 — Supplementary material 1 (DOCX 18 KB) [file 10554_2018_1460_MOESM1_ESM.docx]

**Online Resource 1.** Univariate and multiple linear regression analyses showing possible predictors of absolute stress MBF

|  | Univariate  linear  regression ß (95% CI) | p  Value | R^2^ | Multiple  linear  regression  ß (95% CI) | p Value | R^2^ |
| --- | --- | --- | --- | --- | --- | --- |
| Age | 0.0005 (-0.01 ─ 0.01) | 0.92 | 0.00 |  |  | 0.20 |
| Male gender | -0.49 (-0.69 ─ -0.29) | <0.001 | 0.15 | -0.45 (-0.65 ─ -0.24) | <0.001 |  |
| Hypertension | -0.14 (-0.46 ─ 0.19) | 0.40 | 0.00 |  |  |  |
| Dyslipidaemia | -0.21 (-0.43 ─ 0.01) | 0.06 | 0.03 | -0.20 (-0.40 ─ -0.24) | 0.05 |  |
| DM type 2 | -0.05 (-0.35 ─ 0.24) | 0.72 | 0.00 | -0.02 (-0.04 ─ - 0.002) | 0.08 |  |
| Current smoker | -0.03 (-0.18 ─ 0.12) | 0.69 | 0.00 |  |  |  |
| BMI | -0.02 (-0.04 ─ 0.001) | 0.06 | 0.03 | -0.20 (-0.04 ─ -0.001) | 0.04 |  |
| LVEF in rest | 0.01 (-0.01 ─ 0.03) | 0.07 | 0.03 |  |  |  |
| LVEF in stress | 0.01 (-0.03 ─ 0.03) | 0.12 | 0.01 |  |  |  |
| Calcium score | 0.00 (-0.002 ─ 0.001) | 0.70 | 0.00 |  |  |  |
| Presence of  non-significant  CAD | -0.07 (-0.29 ─ 0.15) | 0.54 | 0.00 |  |  |  |
| LAD-MB | -0.14 (-0.46 ─ 0.19) | 0.40 | 0.01 |  |  |  |

Male gender, dyslipidaemia, DM type 2, and BMI are entered in the multiple linear regression model.
BMI = body mass index; CAD = coronary artery disease; DM = diabetes mellitus; LVEF = left ventricular ejection fraction.
