# Supplementary material for: Myocardial bridging of the left anterior descending coronary artery is associated with reduced myocardial perfusion reserve: a 13N-ammonia PET study
Source: Int J Cardiovasc Imaging. 2018 Sep 28;35(2):375–82. doi: 10.1007/s10554-018-1460-8 (PMC6428791; doi:10.1007/s10554-018-1460-8)
Supplement: Supplementary file 2 — Supplementary material 2 (DOCX 16 KB) [file 10554_2018_1460_MOESM2_ESM.docx]

**Online Resource 2.** Regional comparison of SRS, SSS, and SDS between patients with and without LAD-MB

| Variable | Patients with MB (n=17) | Patients without MB (n=114) | p Value |
| --- | --- | --- | --- |
| **SRS** |  |  |  |
| **LAD** | 0 ( 0 – 1 ) | 0 ( 0 – 0 ) | 0.50 |
| **LCx** | 0 ( 0 – 0 ) | 0 ( 0 – 0 ) | 0.34 |
| **RCA** | 0 ( 0 – 0 ) | 0 ( 0 – 0 ) | 0.14 |
| **SSS** |  |  |  |
| **LAD** | 1 ( 0 – 2 ) | 0 ( 0 – 1 ) | 0.10 |
| **LCx** | 2 ( 0 – 5 ) | 0 ( 0 – 2 ) | 0.04 |
| **RCA** | 1 ( 0 – 4 ) | 0 ( 0 – 2 ) | 0.28 |
| **SDS** |  |  |  |
| **LAD** | 0 ( 0 – 2 ) | 0 ( 0 – 0 ) | 0.14 |
| **LCx** | 2 ( 0 – 5 ) | 0 ( 0 – 2 ) | 0.01 |
| **RCA** | 1 ( 0 – 4 ) | 0 ( 0 – 2 ) | 0.13 |

Values are median and interquartile range.

SSS = summed stress score; SRS = summed rest score; SDS = summed difference score; LAD = left anterior descending artery; LCx = left circumflex artery; MB = myocardial bridging; RCA = right coronary artery.
