# Supplementary material for: Myocardial bridging of the left anterior descending coronary artery is associated with reduced myocardial perfusion reserve: a 13N-ammonia PET study
Source: Int J Cardiovasc Imaging. 2018 Sep 28;35(2):375–82. doi: 10.1007/s10554-018-1460-8 (PMC6428791; doi:10.1007/s10554-018-1460-8)
Supplement: Supplementary file 3 — Supplementary material 3 (DOCX 18 KB) [file 10554_2018_1460_MOESM3_ESM.docx]

**Online Resource 3.** Anatomical characteristics of MB

| Patient | Length (mm) | Depth | Segment of the LAD | Presence of  non-significant CAD |
| --- | --- | --- | --- | --- |
| 1 | 6 | superficial | mid | yes |
| 2 | 7 | superficial | mid | no |
| 3 | 10 | superficial | mid | no |
| 4 | 12 | superficial | mid | no |
| 5 | 14 | superficial | mid | yes |
| 6 | 18 | superficial | mid | yes |
| 7 | 8 | deep | mid | no |
| 8 | 13 | deep | mid | no |
| 9 | 14 | deep | mid | no |
| 10 | 18 | deep | mid | no |
| 11 | 20 | deep | mid | no |
| 12 | 20 | deep | mid | yes |
| 13 | 21 | deep | mid | no |
| 14 | 27 | deep | mid | no |
| 15 | 8 | superficial | distal | yes |
| 16 | 11 | superficial | distal | yes |
| 17 | 13 | deep | distal | no |

MB = myocardial bridging; LAD = left anterior descending artery; CAD = coronary artery disease.
