# Supplementary material for: Myocardial bridging of the left anterior descending coronary artery is associated with reduced myocardial perfusion reserve: a 13N-ammonia PET study
Source: Int J Cardiovasc Imaging. 2018 Sep 28;35(2):375–82. doi: 10.1007/s10554-018-1460-8 (PMC6428791; doi:10.1007/s10554-018-1460-8)
Supplement: Supplementary file 4 — Supplementary material 4 (DOCX 17 KB) [file 10554_2018_1460_MOESM4_ESM.docx]

**Online Resource 4.** Correlations between length and regional quantitative myocardial perfusion in patients with superficial and deep LAD-MB

|  | Length in all MB (n=17) | |
| --- | --- | --- |
|  | Spearman Correlation | P Value |
| Rest MBF (ml/g/min) |  |  |
| Global | -0.14 | 0.59 |
| LAD | -0.13 | 0.61 |
| LCx | -0.15 | 0.56 |
| RCA | -0.08 | 0.75 |
| Stress MBF (ml/g/min) |  |  |
| Global | 0.44 | 0.07 |
| LAD | 0.25 | 0.33 |
| LCx | 0.22 | 0.40 |
| RCA | 0.26 | 0.31 |
| MPR |  |  |
| Global | 0.45 | 0.07 |
| LAD | 0.47 | 0.06 |
| LCx | 0.34 | 0.19 |
| RCA | 0.29 | 0.26 |

LAD = left anterior descending artery; LCx = left circumflex artery; MB = myocardial bridging; MBF = myocardial blood flow; MPR= myocardial perfusion reserve; RCA = right coronary artery.
