# Supplementary material for: Myocardial bridging of the left anterior descending coronary artery is associated with reduced myocardial perfusion reserve: a 13N-ammonia PET study
Source: Int J Cardiovasc Imaging. 2018 Sep 28;35(2):375–82. doi: 10.1007/s10554-018-1460-8 (PMC6428791; doi:10.1007/s10554-018-1460-8)
Supplement: Supplementary file 5 — Supplementary material 5 (DOCX 17 KB) [file 10554_2018_1460_MOESM5_ESM.docx]

**Online Resource 5.** Comparison of quantitative perfusion measurements between deep and superficial MB

| Variable | Deep LAD-MB  (n=9) | Superficial LAD-MB (n=8) | p Value |
| --- | --- | --- | --- |
| **Global Rest MBF (ml/g/min)** | **1.2 ± 0.3** | **1.2 ± 0.3** | **0.87** |
| **LAD** | 1.2 ± 0.3 | 1.2 ± 0.3 | 0.99 |
| **LCx** | 1.3 ± 0.3 | 1.3 ± 0.3 | 0.85 |
| **RCA** | 1.2 ± 0.3 | 1.1 ± 0.3 | 0.74 |
| **Global Stress MBF (ml/g/min)** | **2.2 ± 0.4** | **2.1 ± 0.5** | **0.96** |
| **LAD** | 2.3 ± 0.5 | 2.3 ± 0.5 | 0.91 |
| **LCx** | 2.3 ± 0.5 | 2.3 ± 0.4 | 0.97 |
| **RCA** | 1.8 ± 0.5 | 1.8 ± 0.5 | 0.94 |
| **Global MPR** | **1.8 ± 0.4** | **1.9 ± 0.6** | **0.71** |
| **LAD** | 2.0 ± 0.4 | 2.0 ± 0.5 | 0.99 |
| **LCx** | 1.8 ± 0.4 | 1.8 ± 0.4 | 0.92 |
| **RCA** | 1.6 ± 0.4 | 1.9 ± 1.1 | 0.51 |

Values are mean ± standard deviation.

LAD = left anterior descending artery; LCx = left circumflex artery; MB = myocardial bridging; MBF = myocardial blood flow; MPR= myocardial perfusion reserve; RCA = right coronary artery.
